# Low-Power-Management Engine: Driving DDR Towards Ultra-Efficient Operations

**DOI:** 10.3390/mi16050543

**Published:** 2025-04-30

**Authors:** Zhuorui Liu, Yan Li, Xiaoyang Zeng

**Affiliations:** 1 School of the Academy for Engineering and Technology, Fudan University, Shanghai 200433, China; 19110860029@fudan.edu.cn; 2 The State Key Laboratory of Integrated Chips and Systems, School of Microelectronics, Fudan University, Shanghai 200433, China; xyzeng@fudan.edu.cn

**Keywords:** memory systems, energy efficiency, DRAM controller, bank parallelism, DRAM page policy

## Abstract

To address the performance and power concerns in Double-Data-Rate SDRAM (DDR) subsystems, this paper presents an innovative method for the DDR memory controller scheduler. This design aims to strike a balance between power consumption and performance for the DDR subsystem. Our approach entails a critical reassessment of established mechanisms and the introduction of a quasi-static arbitration protocol for the DDR’s low-power mode (LPM) transition processes. Central to our proposed DDR power-management framework is the Low-Power-Management Engine (LPME), complemented by a suite of statistical algorithms tailored for implementation within the architecture. Our research strategy encompasses real-time monitoring of the DDR subsystem’s operational states, traffic intervals, and Quality of Service (QoS) metrics. By dynamically fine-tuning the DDR subsystem’s power-management protocols to transition in and out of identical power modes, our method promises substantial enhancements in both energy efficiency and operational performance across a spectrum of practical scenarios. To substantiate the efficacy of our proposed design, an array of experiments was conducted. These rigorous tests evaluated the DDR subsystem’s performance and energy consumption under a diverse set of workloads and system configurations. The findings are compelling: the LPME-driven architecture delivers significant power savings of over 41%, concurrently optimizing performance metrics like latency increase by no more than 22% in a high-performance operational context.

## 1. Introduction

In the era of mobile technology growth, compact devices demand expanded performance and energy, highlighting an urgent need for energy-efficient chip design solutions. The memory unit and its access pathways, which serve as the hub for numerous transactions involving various clients, present a prime opportunity for power reduction [1]. In particular, the Double-Data-Rate SDRAM (DDR) system, which has been widely used in modern chips, ranks second with a 23% power-consumption ratio in a modern mobile System on Chip (SoC) [2], emphasizing the importance of innovative solutions to manage DDR system performance and power trade-off effectively.

Previous attempts to address the power-performance issue in DDR systems have been explored. Some have concentrated on hardware architecture enhancements. For instance, the Ambit approach [3] combines a row amplifier with an accelerator for bulk bitwise operations. The MIMDRAM controller solution [4,5] tackles the data movement energy-consumption issue by means of in-DRAM acceleration. Others have utilized Hybrid Memory Cube technology [6], sectored activation along with variable burst length [7], and proposed highly parallel DRAM architectures and FGA techniques to enhance the activation and prefetch energy efficiency [8,9]; these solutions incorporate additional logic modules to improve power efficiency and bandwidth utilization. Notwithstanding these advancements, the high costs related to DRAM manufacturing and the substantial modifications needed in both hardware and software have restricted their extensive application.

In the realm of algorithmic innovation, source-throttling techniques [10,11,12,13] have been proposed to regulate memory request injection rates at the core level. The aim is to mitigate interference by adjusting the request-generation frequency. Additionally, there are efforts to explore the adaptive runtime manager for power-consumption adjustment [14,15,16]. However, these methods primarily operate at the master level and do not fully exploit the internal performance and power characteristics of DRAM. Consequently, the potential of the subsystem remains unoptimized.

Our work differs from these approaches by proposing a new method for the DDR controller scheduler that dynamically enters and exits power-saving modes to enhance DDR power efficiency. At its core, the Low-Power-Management Engine (LPME) uses a statistical analysis of system downtime to dynamically select the most energy-efficient power-saving mode. Our methodology employs a one-tailed Z-test to estimate the probability of data requests during specific timeslots, enabling the selection of the power-saving mode that best suits the system requirements. This approach, with its low computational overhead and innovative modeling technique, provides a precise estimate in the long term, adhering to the law of large numbers. Additionally, the integration of a feedback mechanism helps refine the statistical analysis, preventing sudden changes in power-saving mode recommendations and ensuring a smoother adaptation to varying operational conditions. Our experimental evaluations have shown that in low-power mode, the LPME architecture can achieve a power reduction of over 41% while maintaining performance to result in a latency increase of no more than 22%. In high-performance mode, we saw an average latency reduction of around 11% compared to traditional fixed-timeout methods, significantly improving the DDR subsystem’s performance.

## 2. Foundations of LPME

### 2.1. Theoretical Basis

The foundation of our LPME is predicated on a theoretical model that quantifies power consumption across different operational states. We define $\rho_{i}$ as the power draw of the regular idle state, $\rho_{d}$ as that of the power-saving mode, and $\rho_{a}$ as the energy required to transition from the power-saving mode back to the regular idle state. The core of our power-saving strategy hinges on a simple yet pivotal condition:

(1) $$\left(\rho_{i}-\rho_{d}\right)*tg-\rho_{i}*ta>0$$

**Figure 1:**
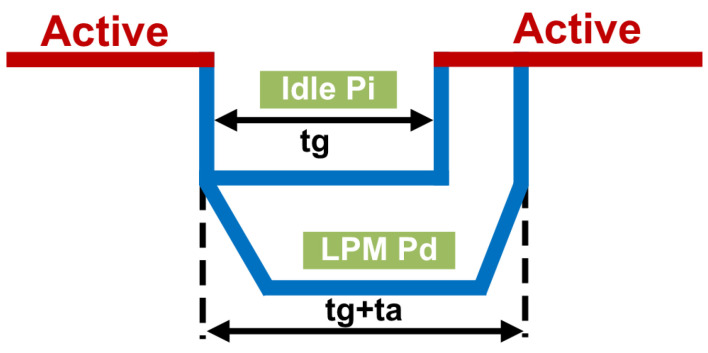
Low-power-mode benefits.

As shown in Figure 1, this inequality suggests that net power savings can be achieved if the gap time tg exceeds the ratio of $\rho_{i}$ to $\rho_{i}-\rho_{d}$. Here, tg symbolizes the gap interval duration, and ta represents the total latency of transitioning back to the idle state. The system’s ability to accurately estimate tg is thus paramount in the decision to engage power saving mode.

### 2.2. Z-Test Application for LPM Control

To address the quantitative analysis of this probability estimate, we employ a Z-test to ascertain the likelihood of data requests within a given time frame, which informs the power-saving-mode selection. The process begins with estimating the expected value, $\rho_{0}$, of the time to the first data request (*T*) under the null hypothesis, with *n* as the trial numbers, and deriving the standard deviation, *s*, as follows:

(2) $$s=\sqrt{\frac{\rho_{0}\left(1-\rho_{0}\right)}{n}}$$

The test’s configuration is determined as one-tailed, given our null hypothesis $H_{0}:\mu >=\rho_{0}$ versus the alternative $H_{1}:\mu <\rho_{0}$. The Z-score is calculated using the number of successful data requests χ over the sample size:

(3) $$Z=\frac{\hat{\chi }-\rho_{0}}{s}\left(\hat{\chi }=\frac{number\;\;of\;\;heads}{sample\;\;size}\right)$$

For a 99% confidence level, the Z-score must meet or exceed 2.58 [17]. This statistical rigor allows us to predict the probability of a “good” slot for power-saving-mode entry, thereby guiding our power management decisions.

### 2.3. Power-Gain Analysis and Decision-Making

Even the Z value will ensure that we have high confidence in identical low-power decisions. In DDR systems, there will be different power modes, which have different power consumption and different enter and exit latency. Therefore, analyzing the power gain ($P_{gain}$) and loss ($P_{loss}$) within fixed time slots is integral to this approach. We denote ts as the slot duration, ta as the latency associated with power-saving mode, and $P_{loss}$ as the power penalty during a wake-up slot. A “good” slot yields a gain of $ts(P_{i}-P_{l})$, where $P_{i}$ is the power in the idle state and $P_{l}$ is the power in the power-saving mode. The decision to enter power-saving mode is predicated on the probability $\rho_{0}$ of a slot being good, aiming for a long-term gain:

(4) $$\rho >\frac{P_{loss}}{P_{gain}+P_{loss}}$$

The selection of an optimal slot-window size ts and the determination of ρ are critical in ensuring a positive power gain, encapsulated by the condition:

(5) $$\rho >\frac{P_{i}}{2P_{i}-P_{l}}$$

With this formula, the $H_{0}$ test for each power-saving mode could be treated as the probability of a “good” slot is greater than $\rho_{0}$ and use 99% as the confidence criteria. This allows us to confidently engage power-saving mode, knowing that with over 99% certainty, the power saved will outweigh the overhead of transitioning states.

Our framework meticulously selects the deepest power-saving-mode level that can be entered based on the DDR subsystem’s idle state. This decision-making process is data-driven, leveraging the power-saving-mode model level to ascertain the most energy-efficient mode upon system idleness. The probability of a “good” slot is greater than $\rho_{0}$ with a 99% confidence criteria. Then, this confidence criteria will drive the system to enter this identical power-saving mode, saving power consumption compared to staying in the active idle state.

In summary, this section establishes the theoretical framework and statistical methodology that underpin our LPME. By combining a rigorous Z-test analysis with a strategic decision-making process, we present a compelling prove for the adoption of our dynamic LPM management strategy, one that promises significant power savings without compromising much system performance.

## 3. Implementation

**Figure 2:**
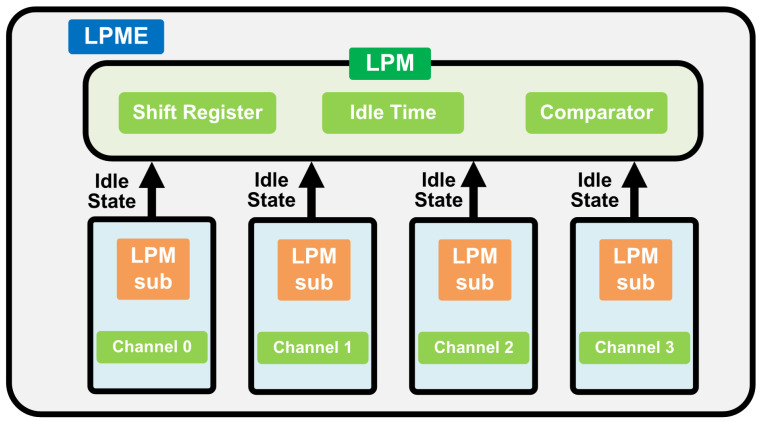
DDR LPME Block Diagram.

As the Figure 2—DDR LPME Block Diagram shows, LPME is the approach used to manage the power modes of both the various DDR sub-channels and a single DDR sub-channel. LPM dynamically recommends the most suitable power-saving modes based on idle time availability. Each DDR channel sends its idle state to the top of the LPM. When all DDR channels are idle and the total number of ‘1’s in the observation window is greater than the threshold for a given power-saving mode, a request is sent to enter the power-saving mode.

As there are different DDR power modes in DDR systems, such as clock idle modes and the deeper power modes, to select the most suitable power-saving mode to enter, the LPM records periods of inactivity, identifying opportunities for deeper LPM engagement. The LPM monitor saves the idle state using a shift register, sampling at every clock. Only idle slots with short durations are shifted into slots. The LPM makes decisions based on the number of ‘1’s in the shift register. The LPM’s decision function is encapsulated by f(ρ)>0, where f(ρ) denotes the expected energy savings per time slot. A Z-test is employed to set thresholds, ensuring that the DDR system’s LPM engagement is both strategic and energy efficient. As shown in Figure 2, this data-driven decision making ensures that the DDR system selects the optimal power-saving-mode level for maximum energy savings without significantly affecting performance.

In addition, the system should not enter power-saving mode with the initial threshold value when waking up from power-saving mode. Therefore, to prevent this, the dynamic threshold should be increased by adding an offset. Similarly, in some situations, the system is active for a long time, and “0” flushes the slots, making it difficult to enter power-saving mode. Therefore, to ensure that the system can enter power-saving mode more quickly, the threshold needs to be decreased to accelerate this process using the adaptive feedback mechanism.

### 3.1. Sampling Mechanism: Detecting Idle States for Power Optimization

**Figure 3:**
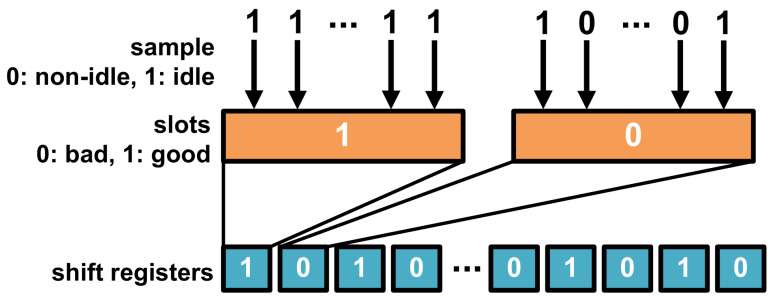
Sampling method of DDR LPM.

Our approach to optimize DDR power consumption includes an enhanced sampling mechanism designed to accurately detect periods of system idleness. By systematically sampling the DDR system at regular intervals, we can precisely determine whether the system is in an idle state, which is characterized by the absence of read or write commands, as shown in Figure 3.

Each sampling period contributes to the formation of a slot, a unit of time within which we aggregate the DDR’s activity status. A slot is classified as “good” if it contains only idle samples, indicating that the DDR was inactive throughout the entire period. This is in contrast to a “bad” slot, which records any activity during the sampling period, thus negating the potential for power savings through LPM engagement. To manage and track the status of each slot orderly, we employ shift registers. These registers provide a reliable and sequential record of whether each slot is “good” or “bad”, offering a clear timeline of the DDR’s idle and active states. This temporal tracking is crucial for our analysis, as it enables us to make informed decisions on the strategic use of power-saving modes to achieve power efficiency.

### 3.2. Multi-Mode Operation: Independent Decision Making in LPM

**Figure 4:**
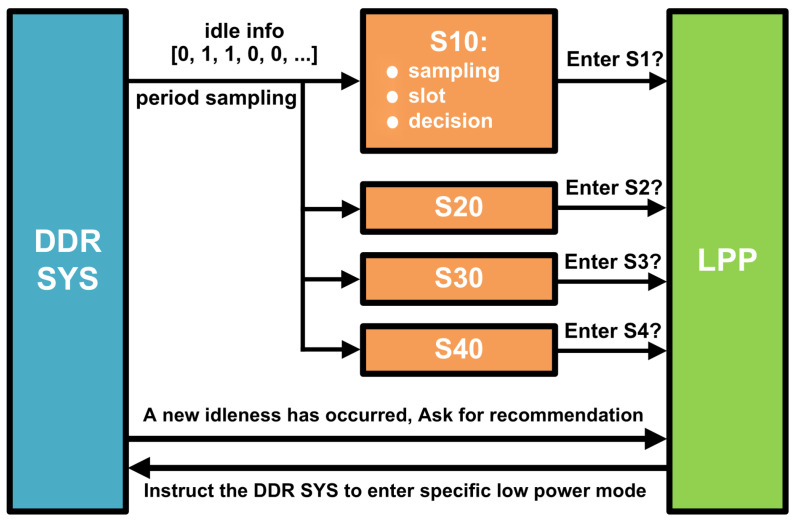
Multi-mode operation of DDR LPM.

In our DDR system architecture, a multitude of LPMs coexist, each monitored by a dedicated Low-Power Observer (LPO). Consider a scenario with four distinct LPMs—S1, S2, S3, and S4—each accompanied by its own observer: S1O, S2O, S3O, and S4O. These observers operate autonomously, making independent decisions based on their unique configurations, as outlined in Figure 4. The process begins with periodic sampling of idle information. Each LPO employs a distinct slot length—comprising a different number of samples—to assess the system’s idle state. The LPOs also utilize varying thresholds to determine the most suitable course of action. The collective recommendations from these observers, suggesting whether to activate a specific power-saving mode, are forwarded to a centralized decision-maker: the Low-Power Planner (LPP). The LPP’s role is pivotal. It evaluates the recommendations and selects the deepest power-saving mode for each channel, with the objective of minimizing power consumption. This strategic selection process is designed to optimize energy use across the DDR system, aligning with our commitment to enhancing overall power efficiency.

### 3.3. Adaptive Feedback Mechanism: Refining LPM Decisions for Optimal Power Management

**Figure 5:**
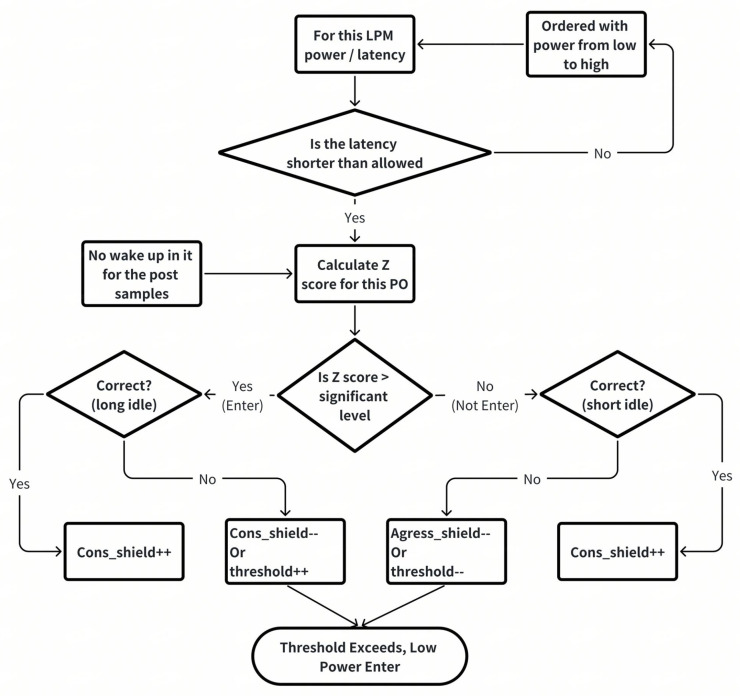
DDR LPME feedback mechnaism.

LPOs within our DDR system use shift registers to meticulously track the status of activity slots, categorizing them as “good” or “bad”. The “good” slots, indicative of idleness, are quantified against a pivotal threshold that guides the decision to enter a low-power mode. When the count of “good” slots surpasses this threshold, the LPO communicates with the LPP, proposing the activation of the corresponding power-saving state. This threshold is dynamic, reflecting the system’s tolerance for incorrect recommendations and adjusting through a sophisticated feedback loop. As depicted in Figure 5, upon detecting a new idle period, the LPM generates a recommendation. Subsequently, after the idleness period, the LPM evaluates the recommendation’s accuracy and adjusts the threshold accordingly. If the recommendation is wrong, the LPM calibrates the threshold to be more conservative or aggressive, ensuring better accuracy in the future.

**Figure 6:**
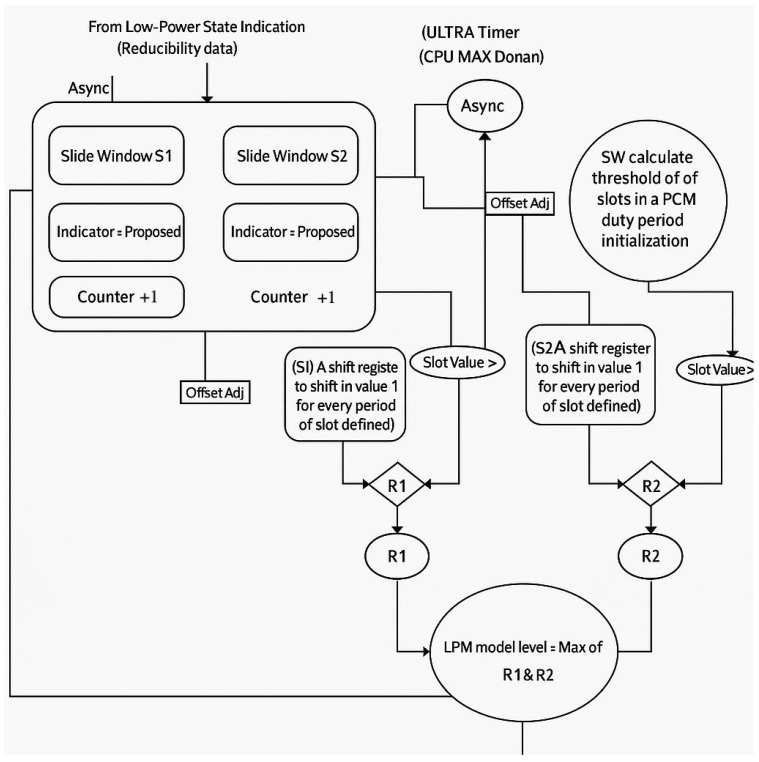
DDR LPM hardware modules.

Conversely, accurate recommendations are met with an increase in a shielding value, which moderates the rate of threshold change, maintaining system stability. This adaptive feedback mechanism is crucial for optimizing power consumption and enhancing overall system performance. By integrating real-time analysis with predictive adjustments as depicted in Figure 6, our DDR system evolves its power-management strategy, becoming more efficient and responsive with each cycle. This intelligent refinement of thresholds and modes represents a significant step towards smarter, more adaptive power-management solutions.

### 3.4. Hardware Complexity

Both the LPME top and LPME sub are implemented near the memory controller and does not introduce any changes to the DRAM chip or its interfaces. We evaluate LPME hardware complexity using Synopsys Design Compiler [18]. We implement LPME in Verilog and synthesize the emitted Verilog HDL design using a Synopsys Design Compiler with a 5nm process technology. DDR and LPDDR share a similar architecture. Their low-power modes include self-refresh mode, low-power standby mode, etc. The difference mainly lies in the power-consumption value, which can be compensated for by different N values. Thus, the impact of configurations can be at the same level.

**Area Analysis.** LPME uses two 32-bit idle counters, one 16-bit threshold counter, and two 1-bit suspect flags per hardware thread, which consume 0.001712 mm^2^ per memory channel at a 5 nm process technology, consuming an overall area overhead of 0.0032% of a state-of-the-art Intel Xeon processor’s chip area [19] (which is implemented in an Intel 10 nm technology node).

**Latency Analysis.** According to our RTL model, LPME can be clocked at 2 GHz (0.5 ns). This latency is faster than the latency of regular memory controller operations as it is smaller than tRRD (e.g., 2.5 ns in DDR4 [20] and 5 ns in DDR5 [21]).

## 4. Evaluation Results

### 4.1. Advantages in Power Efficiency Across Test Scenarios

Our multiple test cases have consistently demonstrated improvements in power efficiency when compared to the no LPM approach. A prime example is the video-recording use case. Utilizing two distinct power-saving modes with entry latencies of 2 ns and 4 ns, respectively, for the same DDR controller and PHY configuration, we observed a remarkable power enhancement exceeding 20% by adopting the power-saving mode, as shown in Table 1.

Upon the activation of the LPM, we noticed a minor average latency increase of 3 ns in the queueing time for individual data requests. This negligible performance impact signifies that the power-saving modes can be effectively engaged without significantly compromising system responsiveness.

### 4.2. In-Depth Analysis of Sampling and Slot-Window Configurations

To validate the effectiveness of the LPME, three typical loading use cases were selected, representing high-bandwidth, medium-bandwidth, and low-bandwidth scenarios. The data patterns for these use cases were generated based on the complex behavior of the SOC. They were formulated to initiate video encoding (VideoEncoder), Douyin-related activities (Douyin), and gaming use cases (Perf joy yuv) within the overall system. The trace data were sourced from the CPU core and the DSA engine, traversing through the bus. Finally, the read and write transactions were input into the DDR controller ports, resulting in bursty and irregular patterns. Further exploration into the nuances of sampling time and slot-window differences revealed a variety of experimental setups with longer low-power-mode enter and exit latency. These included the “Always-small”, “Always-big”, “Idle-small”, and “Fix” methods, each with unique strategies for slot-window adjustments and idle counting mechanisms. The “Always-small” and “Always-big” methods shift the slot window with a slot interval and N slots interval (e.g., N = 50), respectively. The “Idle-small” method shifts the slot window with a slot interval, accounting only for bad slots and disregarding extended good slots. The “Fix” method represents the standard approach to entering power-saving mode, triggering the corresponding state upon reaching a set idle count. The detailed configuration of the LPM systems is derived from modern LPDDR5 subsystems’ DDR low-power-mode entry and exit latencies. The entry time is 134.165 ns, the exit time is 35.361 ns, the power consumption in the low-power mode is 7.92 mW, and the power consumption in the idle mode (when no LPM mode is entered) is 58.18 mW. The number of thresholds is calculated based on Z-test results, which are 43, and the slot number is selected as 50. A larger value of N would provide a more accurate estimate of the average power consumption, but it would also increase the time required to calculate the average. The outcomes of these experiments are meticulously detailed in Table 2.

The results show that even with a fixed option, setting the timeout value to “N” times the minimum level with the sum of the same power-saving-mode entry latency and existing latency is the most aggressive approach for power reduction from the perspective of latency-sensitive use cases like “VideoEncoder” and “Douyin”, but it will increase the overall latency due to many ‘bad’ slots. However, for other interval optimal patterns like “Perfjoy yuv”, the wrong decision number is not so big, and some “good” slots are missed, resulting in suboptimal power consumption.

Finding a fixed value to meet all use cases’ requirements is not possible, as these use cases switch automatically and DDR settings are fixed after initialization. This traditional fixed power-saving-mode method cannot track pattern behavior like the other three options. Among the “always-small”, “always-big”, and “idle-small” options, “idle-small” outperforms in energy savings and “always-small” outperforms in latency increase. Besides the algorithm contribution, “idle-small” only tracks the bad slots, which makes this sampling method more aggressive than the other two “always” methods.

Furthermore, the shifting logic can be clock gated when continuous good slots occur, saving more power than the other two methods. However, this is not the optimal way, as when continuous bad slots occur, the timeout value decreases sharply, causing more incorrect slots to occur. To solve this problem, we further examined the dynamic tuning method, as shown in the next subsection.

### 4.3. Dynamic Tuning and Configuration Selection for Optimal Results

**Figure 7:**
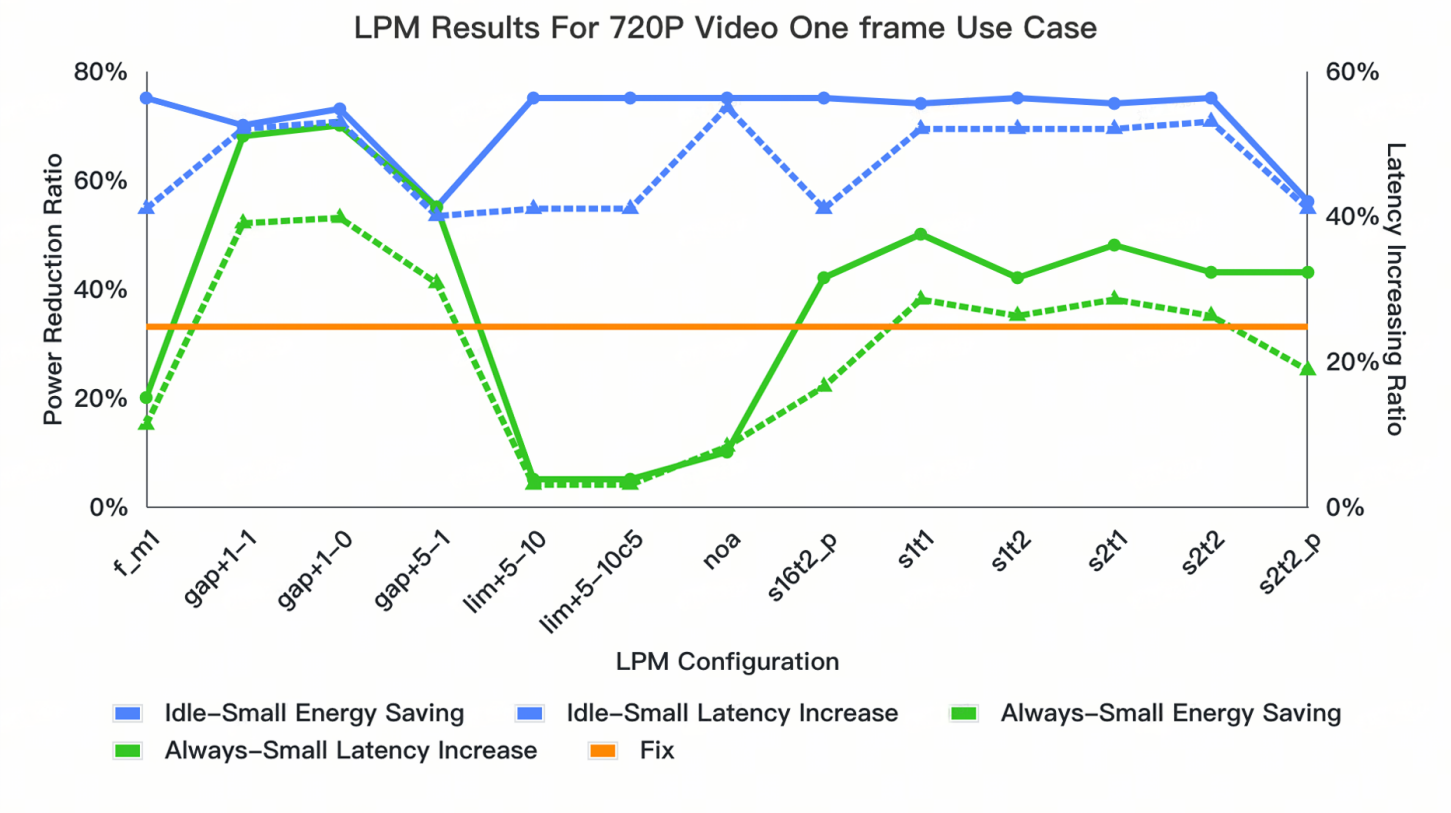
Comparison results of different LPM configurations of 720p Douyin with fixed mode.

**Figure 8:**
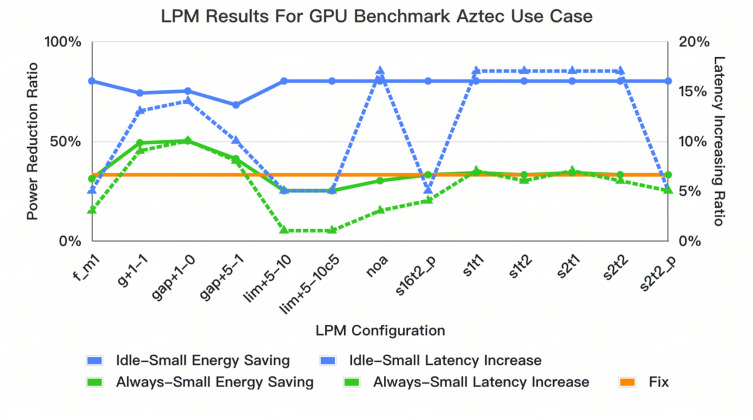
Comparison results of different LPM configurations of GPU Benchmark Aztec with fixed mode.

In the Figure 7 and Figure 8, we present detailed results for dynamic tuning. These results visually compare the performance differences between the dynamic LPM configurations and the fixed-timeslot methodology. We compare two dynamic sampling methodologies, gap*, lim*, and s*t* (“*” is the step granularity; “+” means forwarding direction; “−” means the oppisite), with static setting to ensure a fair comparison. The dynamic method allows for shielding adjustments that improve energy efficiency, avoiding aggressive entries into power-saving modes that can lead to incorrect decisions. For example, the always-small configuration is ideal for latency reduction, reducing it by around 2% compared to static methods in these four use cases; using a lim+5−10 configuration with the idle-small setting ensures power reduction, achieving an average 14% latency reduction. The s1t* also reduces the latency and maintains the power reduction with less prediction error. This provides a more flattened and optimal dynamic tuning method, balancing energy saving and latency loss compared to the NOA options.

## 5. Conclusions

The LPME achieves significant energy savings in the most common LPDDR5 subsystems that are employed in mobile devices for typical multimedia-related use cases and sets a precedent for future innovations in dynamic power management. It achieves a power reduction of 41% across various multimedia test cases such as the 720P Video One-Frame Use Case and GPU Benchmark Use Case with power-saving intent, accompanied by a latency optimization of around 14% compared to the traditional “fix” modes described in the previous chapter, which represent the state-of-the-art (SOTA) commercial DDR controller, and furthermore, in comparison with prior techniques like MIMDRAM or Ambit, our work does not require a modification of the DRAM device itself. This not only saves a significant amount in costs but also enables easy integration with the DDR controller. In addition to the dynamic tuning across diffferent use cases, for one single use case(GPU Benchmark Use Case), we can also set the LPM configuration to the dynamic tuning mode setting discussed in Section 4.3 to eliminate the latency increase by no more than 22%. Thus, with the dynamic setting tuning method, the results underscore the importance of balancing power savings with performance based on specific application requirements. However, the trade offs, such as worst-case latency and system stability during rapid power-state changes, still require other components of DDR controller techniques for handling. For instance, the transaction time-out mechanism is needed to maintain the worst-case latency, and for the row hammer issue, techniques like PRAC (Programmed Row Activation) and RFM (Row Hammer Mitigation) are relevant. From a software implementation viewpoint, the LPME engine could be integrated with commercial memory controllers directly with a standard DDR low-power-mode enter and exit interface with SOC, which facilitates the usage of this technique. Overall, with a straightforward Z-score calculation and potential for software implementation, the LPME represents a practical advancement towards smarter, more efficient DDR systems.

## Figures and Tables

**Table 1:** LPM power benefit.

Method	No LPM	LPM	Ratio
Total Transaction Time (ns)	299,491	299,495	0.01%
Bandwidth (MB/s)	6338.96	6338.88	0.01%
Total Power (mw)	121.33	94.26	−22%

The ratio in Table 1 refers to the ratio of the power consumption of the LPME to the power consumption of the traditional method. The ratio is calculated by dividing the power consumption of LPME by the power consumption of the traditional method. The value is less than 1, which means that LPME consumes less power than the traditional method.

**Table 2:** Slot-window selection benefit.

Case	Algorithm	PR Ratio ^a^	PE Ratio ^b^	Idle ^c^
VideoEncoder	fixed	16.78	7.2	37,514
Douyin	fixed	15.83	7.33	29,848
Perf joy yuv	fixed	52.03	5.02	17,678
VideoEncoder	always-small	9.68	3.85	82,586
Douyin	always-small	4.21	3.22	53,681
Perf joy yuv	always-small	66.97	11.49	14,498
VideoEncoder	always-big	9.33	4	82,405
Douyin	always-big	4.05	3.45	53,324
Perf joy yuv	always-big	66.75	11.7	3860
VideoEncoder	idle-small	9.68	4.28	84,912
Douyin	idle-small	4.05	6.84	56,572
Perf joy yuv	idle-small	69.73	10.67	12,946

^a^ “PR Ratio” means the ratio of energy saved compared with no power-saving mode enabled; the unit is ‘%’. ^b^ “PE Ratio” means the latency-increased ratio with no power-saving mode enabled; the unit is ‘%’. ^c^ “Idle” means during the overall use cases’ duration, which is the number observed when the engine is in an idle state.

## Data Availability

The code will be open source after the paper is accepted.
